# Comparisons of statistical distributions for cluster sizes in a developing pandemic

**DOI:** 10.1186/s12874-022-01517-9

**Published:** 2022-01-30

**Authors:** M. J. Faddy, A. N. Pettitt

**Affiliations:** School of Mathematical Sciences and ARC Centre of Excellence for Mathematical and Statistical Frontiers, QUT, GPO Box 2434, Brisbane, 4001 Australia

**Keywords:** Cluster size, COVID-19, Extended Poisson process, Negative binomial distribution, Superspreading event

## Abstract

**Background:**

We consider cluster size data of SARS-CoV-2 transmissions for a number of different settings from recently published data. The statistical characteristics of superspreading events are commonly described by fitting a negative binomial distribution to secondary infection and cluster size data as an alternative to the Poisson distribution as it is a longer tailed distribution, with emphasis given to the value of the extra parameter which allows the variance to be greater than the mean. Here we investigate whether other long tailed distributions from more general extended Poisson process modelling can better describe the distribution of cluster sizes for SARS-CoV-2 transmissions.

**Methods:**

We use the extended Poisson process modelling (EPPM) approach with nested sets of models that include the Poisson and negative binomial distributions to assess the adequacy of models based on these standard distributions for the data considered.

**Results:**

We confirm the inadequacy of the Poisson distribution in most cases, and demonstrate the inadequacy of the negative binomial distribution in some cases.

**Conclusions:**

The probability of a superspreading event may be underestimated by use of the negative binomial distribution as much larger tail probabilities are indicated by EPPM distributions than negative binomial alternatives. We show that the large shared accommodation, meal and work settings, of the settings considered, have the potential for more severe superspreading events than would be predicted by a negative binomial distribution. Therefore public health efforts to prevent transmission in such settings should be prioritised.

**Supplementary Information:**

The online version contains supplementary material available at 10.1186/s12874-022-01517-9.

## Background

In the public communication of information about the novel coronavirus disease COVID-19 there is emphasis frequently given to estimates of the basic reproduction number *R*_0_, the expected number of secondary cases arising from a single primary case in a fully susceptible population, and the effective reproduction number *R*_*t*_ which is similarly defined to *R*_0_ but when a population is subject to control measures. Values of such *R*’s greater than one lead to the exponential growth of daily case numbers.

Of next importance, or perhaps equal, to the value of *R*_0_ or *R*_*t*_ is a measure of individual variation in infectiousness. Such variation is important because it can explain the occurrence of so-called superspreading events (SSE’s) where the numbers of secondary and subsequent cases are substantially more than expected from assuming a Poisson distribution for the numbers of cases. In a Nature News Feature [1] the importance of SSE’s in the continued COVID-19 pandemic is emphasized and relevant research reviewed.

SSE’s were considered in [2] by comparing right tail probabilities of the Poisson distribution with alternatives for the distribution of secondary cases. For highly skew negative binomial distributions, with variance equal to mean + mean^2^/*k* and small values of the extra parameter *k*, the tail probabilities are much larger than those for the corresponding Poisson distribution with the same mean. The paper emphasises the importance of recognising the reasonable chance of SSE’s in the effective control of infectious diseases by targeting individual-specific control measures rather than population wide measures.

The importance of SSE’s in the COVID-19 pandemic is shown in [3] where the transmission of the virus SARS-CoV-2 is characterised by high stochasticity under low prevalence. The importance of the control of SSE’s in the transmission of SARS-CoV-2 is stressed, and various types of SSE are described using negative binomial tail probabilities with small dispersion parameter *k* to quantify the probability of an SSE.

Genomic epidemiology was used [4] to investigate the transmission of SARS-CoV-2 in the Boston, MA, area concluding that not all SSE’s for COVID-19 have the same impact on the community. Two SSE’s were compared. One, in a skilled nursing facility led to widespread transmission within the facility, while another, in an international business conference, had a much greater impact on the local, US and international community with a very large number of transmissions. This study does suggest that SSE’s can affect in diverse ways the course of an epidemic, and that prevention, detection, and mitigation of such events should be a priority for public health efforts.

Given data on the number of secondary cases arising from each primary case, either individual secondary case data or clusters of such data, then the epidemiological literature has suggested using the negative binomial distribution as an alternative to the Poisson distribution. Cluster size includes all primary or initial cases, secondary cases and all subsequent derived cases. This distribution was considered [2] for the number of secondary cases for a number of infectious diseases including severe acute respiratory syndrome (SARS). A formula was derived for the probability of a given cluster size given a known number of initial cases, and the dispersion parameter *k* was estimated to be 0.16 with 90% confidence interval (0.11, 0.64) from data on the Singapore SARS outbreak due to the SARS-CoV-1 virus. The negative binomial and geometric distributions were favoured over the Poisson distribution for several disease datasets.

For Middle East respiratory syndrome coronavirus (MERS-CoV) data clusters of cases from around the world were considered in [5], and a negative binomial distribution for the number of secondary cases was fitted and estimates reported for MERS-CoV: *k* = 0.26 (90% CI: 0.11, 0.87, 95% CI: 0.09, 1.24).

For COVID-19 data sets, the negative binomial distribution was fitted to sizes of outbreaks or clusters outside China in [6]. A dispersion parameter *k* estimate of 0.1 was reported, with 95% Credible Interval (0.05, 0.2) for *R*_0_ equal to 2.5 (thereby ignoring any uncertainty in the estimation of *R*_0_). Joint inference for both *R*_0_ and *k* does have a large degree of uncertainty, but even so using WBIC it was shown that the negative binomial distribution was greatly favoured over the Poisson distribution.

The zero-truncated negative binomial distribution was fitted to COVID-19 secondary case data in [7], including possible cluster sizes of one; that is, cases with no further infections. The parameter *k* was estimated to be 0.32, 95% CI (015, 0.64).

Given the common use of the negative binomial distribution to model the distribution of secondary cases, cluster sizes and hence SSE’s, it is important and of interest to investigate whether other long tailed distributions can reasonably describe the distribution of secondary cases and cluster sizes for SARS-CoV-2 transmissions and whether these distributions lead to more extreme SSE’s than predicted by a negative binomial distribution.

## Methods

### Data

The data analysed in this paper are from an Excel database dated 06-07-2020 (North American style) [8]. They were collected for various “settings” within which infections took place; these ranged from “Building site” to “Work”. The database gave “The total number of cases per cluster” for all 265 entries, with breakdowns into numbers of “primary cases” and” secondary cases” only included for less than a third of these. So to maximize the amount of data available for analysis, cluster sizes were considered as the response with settings as a covariate. Transmission of infection was presumed to be limited to the specific setting with no infections from other settings included.

The overall aim in [8] was to gather information on reported clusters of COVID-19 cases to determine the different settings in which SARS-CoV-2 transmission was occurring. This was done from a search of the scientific literature and media articles detailing clusters of SARS-CoV-2 transmission. Data from such sources will have several limitations, including inherent recall bias, biased media reporting and incomplete information – deciding to work with data on cluster sizes being a consequence of the last of these. About half the data related to outbreaks occurring in China and Singapore.

### Modelling

The Poisson distribution ([9], chapter 4) is a basic statistical model for count data and corresponds to “events” occurring randomly over time in a Poisson process of fixed “rate”, *λ* > 0; that is, where the probability of an event occurring in a short time interval of length *δt* is *λδt*, independently of the occurrence of any other events. The number of events occurring in a finite time interval of length *t* (which can be taken, without loss of generality, to be one) then has the Poisson distribution with mean = variance = *λ*. This limitation on the variation has prompted generalisations to admit variance greater than the mean with the negative binomial ([9], chapter 5) being one such generalisation where the rate of the Poisson process is allowed to vary between different observed processes according to a gamma distribution with shape parameter *k* (> 0); this leads to a mixed Poisson distribution with variance = mean + mean^2^/*k*. Such a measure, *k*, of extra variation compared to Poisson in any mixed Poisson distribution expressed generally as *μ*^2^/(*σ*^2^ - *μ*), where *μ* and *σ*^2^ are the mean and variance of the counts, is the reciprocal of the square of the coefficient of variation of the mixing distribution. So the smaller this quantity is the greater the extra variation with the Poisson distribution corresponding to an infinite value.

An alternative way of varying the rate of the underlying Poisson process is to relax the above assumption of independence and have dependency on the accumulating number of events *n*, say, occurring: *λ*_*n*_ = *a*(*n* + *k*), *n* ≥ 0, corresponds to the negative binomial distribution with mean *k* [exp(*a*) - 1]. Having *λ*_*n*_ = *a*(*n* + *b*)^*c*^ with *c* ≠ 1 thus generalises the negative binomial distribution (*c* = 1 and *b* = *k*) with *c* < 0 resulting in variance < mean, and *c* > 0 variance > mean [10]. So this generalisation admits a range of dispersion from sub-Poisson (*c* < 0) through Poisson (*c* = 0) to negative binomial (*c* = 1) and beyond (*c* > 1). Details of calculating the probabilities from sequences *λ*_*n*_, *n* ≥ 0, are given in an Additional file 1.

This extended Poisson process modelling (or EPPM) was introduced in [11] with subsequent papers ([12] and references therein) developing and applying this modelling. A complication arises when the power parameter *c* in the above formulation of the *λ*_*n*_’s exceeds one, in that the resulting probability distribution is *dishonest* with the sum of the probabilities being less than one, and they require re-normalising by dividing by this sum before they can be applied – details of doing this in practice are also given in the Additional file 1.

Using this *λ*_*n*_ = *a*(*n* + *b*)^*c*^, *n* ≥ 0, formulation a single degree of freedom test for departures from negative binomial variation thus corresponds to testing the hypothesis *c* = 1 (with *b* = *k*). Although *c* = 0 corresponds to *λ*_*n*_ being constant and the Poisson distribution, the parameter *b* becomes redundant in this special case. However, the limiting case of *b* → ∞ and *c* → ±∞ leads to *λ*_*n*_ = *α* exp. (*βn*) with *β* = 0 corresponding to the Poisson distribution, and *β* > 0 (< 0) to probability distributions with variance >(<) mean (again, re-normalisation of the probabilities is required if *β* > 0). Hence a single degree of freedom test for departures from the Poisson distribution can be carried out.

### Model fitting

All the probability distributions considered:

(i) basic Poisson distribution with variance = mean,
(ii) EPPM with *λ*_*n*_ = *α* exp. (*βn*), *n* ≥ 0 (to assess the adequacy of the Poisson distribution, *β* = 0),
(iii) negative binomial distribution (if Poisson is inadequate, to estimate the additional dispersion parameter *k*), and
(iv) EPPM with *λ*_*n*_ = *a*(*n* + *b*)^*c*^, *n* ≥ 0 (to assess the adequacy of the negative binomial, *c* = 1)

Have support on 0, 1, 2, … but cluster sizes are by definition non-zero. Distributions (i) – (iv) were therefore fitted to cluster sizes minus one for each setting. Maximum likelihood estimation (again, practical details including MATLAB® code are in the Additional file 1), rather than simple moment estimation, was used in fitting the distribution and the results compared across the different settings. For each setting, a Poisson model was first fitted to the data and compared with an EPPM (ii) fit using a likelihood ratio test; if there were no significant improvement then the Poisson model was selected. If there were significant improvement, then a negative binomial model was fitted and compared with an EPPM (iv) fit; if this resulted in no further significant improvement in fit then the negative binomial model was chosen. If there were some significant improvement, then the EPPM (iv) model was selected; since this was the most general of the models, assessments of the resulting fits were done using P-P plots.

## Results

**Fig. 1:**
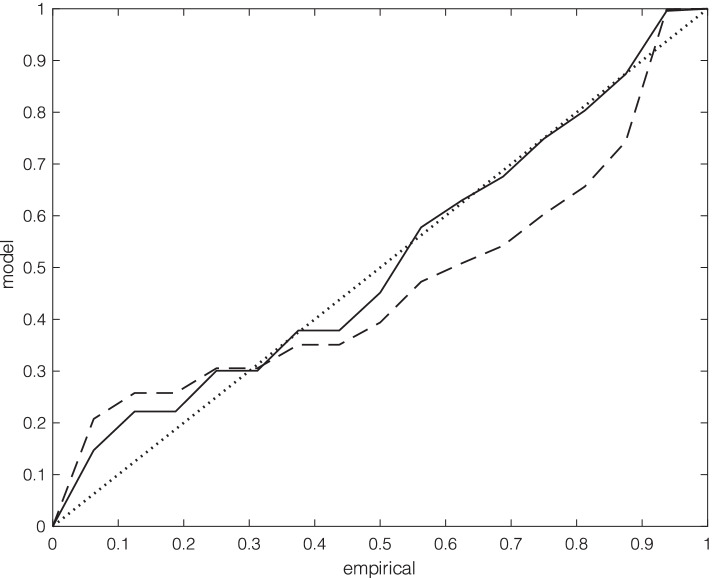
P-P plots for fitted models to the work data: negative binomial (dashed line) and EPPM (solid line); the diagonal dotted line represents perfect correspondence

The data [8] provided ten subsets of specific settings of SARS-CoV-2 transmission, where there were sufficient numbers of clusters (at least 10) for reasonable prospects of getting Sufficiently precise estimates. These were: elderly care (*n* = 21), food processing plants (*n* = 21), household (*n* = 38), large shared accommodation (*n* = 29), meal (in restaurants, etc., *n* = 17), party (*n* = 14), religious (gatherings, *n* = 22), school (*n* = 11), sport (*n* = 22) and work (*n* = 15). Table 1 shows results from the above fitting process, with the most parsimoniously parameterised model chosen. The P-P plots in Fig. 1 show the improvement in fit of the EPPM (iv) over the negative binomial model for the work data, with the former showing quite a good fit to the data and a better match to the upper tail of the empirical distribution. The models fitted to the large shared accommodation and meal data showed similar P-P plots.

**Table 1:** Details of model fitting for ten different settings for COVID-19 cluster size data [8]. One of three possible models [(i) – (iv), as described in the text] was chosen based on the log-likelihood ratio statistic, the value of which is given for testing a simpler model versus a more complex one [*β* = 0 for (ii) versus (i), and *c* = 1 for (iv) versus (iii)]. The *p*-value of this statistic is also given

Setting	Model chosen	Log-likelihood ratio statistic for comparison with alternative model	*p*-value for comparison with alternative model
Elderly care	Negative binomial	1.54	0.21
Food processing plants	Negative binomial	0.25	0.62
Household	Poisson	2.30	0.13
Large shared accommodation	EPPM	5.18	0.023
Meal	EPPM	7.19	0.0073
Party	Negative binomial	0.44	0.51
Religious	Negative binomial	0.12	0.73
School	Negative binomial	0.53	0.47
Sport	Negative binomial	1.15	0.28
Work	EPPM	10.00	0.0016

Only household transmission could reasonably be described by the Poisson distribution (chi-squared goodness of fit statistic 2.21 on 4 d.f.), with all the other settings showing highly significant departures from Poisson variation (*p*-values < 0.001). The negative binomial distribution was quite adequate for elderly care, food processing plants, party, religious, school and sport. However, large shared accommodation, meal and work all showed significantly more variation than that described by the negative binomial distribution.

The maximum likelihood estimates of the parameter *k* from the negative binomial fits to the above mentioned six subsets varied between 0.66 with standard error 0.17 (food processing) and 1.16 with standard error 0.34 (elderly care). These relatively large standard errors suggest that this parameter may be assumed to be constant across the subsets, which is confirmed by fitting negative binomial distributions with the same value of the *k* parameter but different values of the means to these data: log-likelihood ratio statistic 2.75 on 5 d.f. Shown in Table 2 are the estimated means and (common) parameter *k*.

**Table 2:** Details of parameter estimates with standard errors (s.e.) for ten different settings for COVID-19 cluster size data [8]. Values are given for the dispersion parameter, or *k*-parameter, defined for all models fitted and the *c*-parameter for the EPPM models. A value of 0 for the *c*-parameter corresponds to a Poisson model and a value of 1 a negative binomial model

Setting	Mean estimate (s.e.)	*k*-parameter estimate (s.e.)	*c*-parameter estimate from EPPM formulation (s.e.)
Elderly care	37.95 (9.01)	0.86 (0.11)	1
Food processing plants	187.47 (44.10)	0.86 (0.11)	1
Household	3.68 (0.60)	∞	0
Large shared accommodation	85.19 (37.23)	0.23 (0.29)	1.24 (0.13)
Meal	6.03 (1.53)	0.64 (0.34)	3.97 (1.19)
Party	29.14 (8.50)	0.86 (0.11)	1
Religious	39.77 (9.22)	0.86 (0.11)	1
School	33.91 (11.13)	0.86 (0.11)	1
Sport	5.86 (1.44)	0.86 (0.11)	1
Work	11.62 (3.04)	0.42 (0.24)	3.34 (0.76)

Estimates of the parameter *k* for the large shared accommodation, meal and work subsets had to be calculated from first principles from the fitted distributions, as there are no simple algebraic expressions for the moments from the EPPM formulation; this does make likelihood based inferences problematic. The three estimates of *k* and their standard errors are also shown in Table 2; all three estimates are lower than the (common) estimate of *k* for the other settings (excluding household). However, the standard errors are very large, suggesting that the three subsets having the same value of *k* could be a quite acceptable hypothesis, but also that *k* might not be such a useful measure of extra variation for long-tailed distributions like EPPM’s,.

**Fig. 2:**
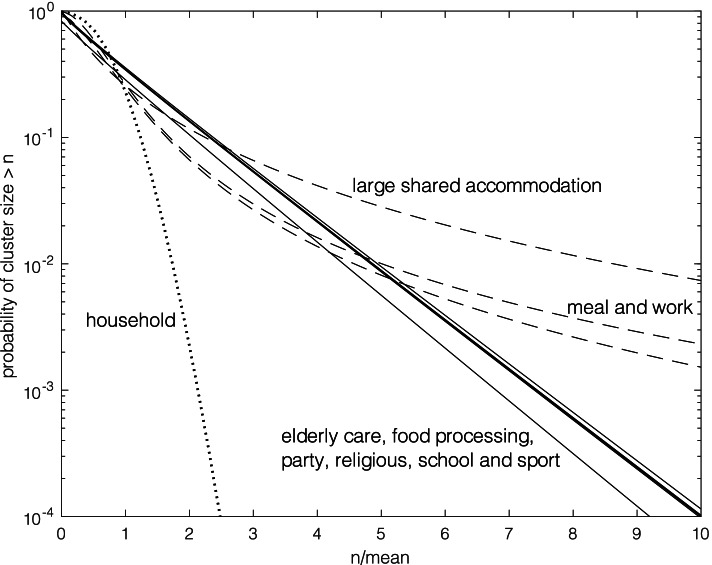
Shows plots of the fitted probability distributions corresponding to the estimated models described in Tables 1 and 2 for COVID-19 cluster size data [8]. These are of upper tail probabilities of cluster size plotted against multiples of their respective means. Poisson (dotted line), negative binomial (solid lines) and EPPM (dashed lines)

In Fig. 2 are plots of the fitted probability distributions corresponding to the estimated models described in Tables 1 and 2. As the means varied from about four (household) to nearly 200 (food processing), upper tail probabilities have been plotted against multiples of their respective means. The negative binomial distributions (elderly care, food processing.

plants, party, religious, school and sport) all with a common value of the parameter *k* are quite similar showing a roughly linear decline on the log-scale. The EPPM distributions for.

Meal and work with lower values of *k* are also quite similar but convex decreasing (on the log-scale) indicating the much longer tails of these distributions. While the EPPM distribution for large shared accommodation with an even lower *k* and also a lower value of the parameter *c* shows even longer tails. The Poisson (household) distribution shows its much shorter tail with a concave decreasing plot.

## Discussion

Maximum likelihood estimation has been used here to estimate the dispersion parameter *k*, rather than moment estimation which seems to be more common in epidemiological studies [2, 5–7]. Indeed, the estimates quoted in Table 2 are somewhat larger than those from these studies, although they are all rather imprecise (with wide confidence intervals or large standard errors). Estimates based on moments, or *k*-statistics, do have a tendency to be smaller with data from long-tailed probability distributions as simple moments are more influenced by extremes than maximum likelihood estimates.

Even though, as shown in Fig. 2, larger tail probabilities of the negative binomial compared to Poisson are plainly apparent, superspreading may be underestimated by use of the negative binomial distribution [2, 3] as much larger tail probabilities are indicated from the EPPM distributions.

Cluster sizes are necessarily positive and have been modelled using truncated (zero excluded) distributions with support on 1, 2, … in [7]. Here, untruncated distributions have been used to model cluster size minus one which has an interpretation as the number of additional infections in a superspreading event initiated by a single infected individual. Both truncated and untruncated modelling gave similar results from the data considered here, with no one type consistently preferred over the other by likelihood based criteria. However, untruncated EPPM’s were preferred over truncated for the large shared accommodation, work and meal data.

Extreme value like distributions offer other longer-tailed alternatives to the negative binomial distribution, but the theory behind such distributions (i.e., maxima of equal sample sizes) would not apply to epidemic clusters. Pragmatically though, distributions of such forms could be applied to any data but they should be in discrete form for numbers of infections. For example, a discrete Pareto distribution with probability mass function proportional to (*n* + *a*)^-(*b* + 1)^, for *n* = 0, 1, 2, … with *a*, *b* > 0, gave much the same fits to the large shared accommodation and work data as the EPPM’s, but a rather worse fit to the meal data – much the same as the negative binomial in fact. Such distributions are quite different from EPPM’s, without the Poisson and negative binomial distributions as special cases. And the EPPM family of distributions does have the appeal of containing these standard distributions as special cases enabling significance testing of improvements from more general alternatives within the family.

Settings of SARS-CoV-2 infection have been the subject of other recent studies. In [13] the authors investigated the risk of transmission in outdoor settings compared with indoor settings, conducting a systematic review of peer-reviewed papers and identifying five studies which found a lower proportion of reported global SARS-CoV-2 infections occurring outdoors: odds-ratio of 18.7 (95% confidence interval 6.0–57.9) for infection indoors compared with outdoors. And in [14] the authors analysed outbreaks by industry sector in the first wave of the pandemic, and associated household cases. They found that 80% of cases belonged to three sectors: manufacturing; agriculture, forestry, fishing and hunting; and transportation and warehousing. Household cases were associated with 31% of outbreak cases, increasing the burden of illness by 56%. The results presented here are not dissimilar in that SSE’s were associated with the work and meal settings, the latter being an indoor setting as would be large shared accommodation. And households being associated with increasing the burden of illness would not be at variance with the finding here of household infection not initiating SSE’s per se.

## Conclusions

Long-tailed probability distributions are necessary to adequately describe the variation in sizes of clusters of infections emanating from a common source, as the Poisson distribution was quite inadequate for all but one of the subsets of data considered. The negative binomial distribution proved to be adequate for all but three of the other settings, where the extra parameter of the EPPM formulation was necessary to describe the variation. Given that the negative binomial distribution was indicated for 60% of the settings considered here, this model might be considered as a “standard” for numbers of infections in a developing pandemic, with the Poisson (less dispersion) as well as the EPPM (greater dispersion) notable exceptions. Implications of such longer-tailed distributions of numbers of infections in more complex modelling of epidemics would be of further interest.

Considerable heterogeneity of transmission is apparent with large shared accommodation, meal and work having higher excess variation and being the most heterogeneous, while household was more homogeneous with the Poisson distribution adequate. Household also had the lowest mean cluster size, with food processing the highest. The large shared accommodation, meal and work settings, of those considered, have the potential for more severe SSE’s than would be predicted by a negative binomial distribution, which suggests that public health efforts to reduce transmission in such settings (such as encouraging working from home wherever possible, for example) should be a high priority.

The data considered in this paper were aggregated from many countries in Asia, Europe and North America, and so are certainly comprehensive; they are openly available and their sources varied. They are probably as reliable as any data on a developing pandemic, and adequately accounting for their variability can only be beneficial to the robustness of any inferences.

## Supplementary Information

**Additional file 1.**

## Data Availability

The dataset analysed is available from https://bit.ly/3ar39ky

## Abbreviations

EPPM: Extended Poisson process model (ling)
s.e.: Standard error
SSE: Superspreading event
WBIC: Widely applicable Bayesian information criterion

## References

[1] Lewis D (2021). Superspreading drives the COVID pandemic – and could help to tame it. Nature..

[2] Lloyd-Smith JO, Schreiber SJ, Kopp PE (2005). Superspreading and the effect of individual variation on disease emergence. Nature..

[3] Althouse BM, Wenger EA, Miller JC (2020). Superspreading events in the transmission dynamics of SARS-CoV-2: opportunities for interventions and control. PLoS Biol.

[4] Lemieux JE, Siddle K, Shaw BM (2021). Phylogenetic analysis of SARS-CoV-2 in Boston: highlights the impact of superspreading events. Science..

[5] Kucharski AJ, Althaus CL (2015). The role of superspreading in Middle East respiratory syndrome coronavirus (MERS-CoV) transmission. Euro Surveil.

[6] Endo A, Abbott S, Centre for the Mathematical Modelling of Infectious Diseases COVID-19 Working Group (2020). Estimating the overdispersion in COVID-19 transmission using outbreak sizes outside China. [version 3; peer review: 2 approved]. Wellcome Open Res.

[7] Zhao S, Shen M, Musa S (2021). Inferencing superspreading potential using zero-truncated negative binomial model: exemplification with COVID-19. BMC Med Res Methodol.

[8] Leclerc QJ, Fuller NM, Knight LE (2020). What settings have been linked to SARS-CoV-2 transmission clusters? [version 2; peer review: 2 approved]. Wellcome Open Res.

[9] Johnson NL, Kemp AW, Kotz S (2005). Univariate discrete distributions.

[10] Ball F (1995). A note on variation in birth processes. Math Sci.

[11] Faddy MJ (1997). Extended Poisson process modelling and analysis of count data. Biom J.

[12] Smith DM, Faddy MJ (2016). Mean and variance modelling of under- and over-dispersed count data. J Stat Softw.

[13] Bulfone TC, Malekinejad M, Rutherford GW (2021). Outdoor transmission of SARS-CoV-2 and other respiratory viruses: a systematic review. J Infect Dis.

[14] Murti M, Achonu C, Smith BT (2020). COVID-19 workplace outbreaks by industry sector and their associated household transmission, Ontario, Canada, January to June, 2020. J Occup Environ Med.

