# Supplementary material for: Comparisons of statistical distributions for cluster sizes in a developing pandemic
Source: BMC Med Res Methodol. 2022 Jan 30;22:32. doi: 10.1186/s12874-022-01517-9 (PMC8801190; doi:10.1186/s12874-022-01517-9)
Supplement: Supplementary file 1 — Additional file 1. [file 12874_2022_1517_MOESM1_ESM.docx]

**Appendix**

The basic extended Poisson process model (EPPM) is a pure birth process {*X*(*t*); *t* ≥ 0} over time *t* with *X*(0) = 0 and transition probabilities:

P{*X*(*t* + *δt*) = *n* + 1 | *X*(*t*) = *n*} = *λ_n_δt* + o(*δt*)

for *n* = 0, 1, 2, … where *λ*_0_, *λ*_1_, *λ*_2_, … form a sequence of (birth) rate parameters. These lead to probabilities:

[P{*X*(*t*) = 0} P{*X*(*t*) = 1} … P{*X*(*t*) = *N*}] = [1 0 … 0] exp(***Q****t*)

where ***Q*** is a (*N*+1) × (*N*+1) bi-diagonal matrix with −*λ*_0_, −*λ*_1_, …, −*λ_N_* on the diagonal and *λ*_0_, *λ*_1_, …, *λ_N_*_-1_ on the upper diagonal, and *t* can be taken without loss of generality to be 1. Here *N* is arbitrary to give a finite dimensional matrix ***Q*** so that the matrix exponential exp(***Q***) can be calculated numerically from routines that are available in packages such as MATLAB^®^.

The likelihood of data *y*_1_, *y*_2_, …, *y_n_* is then P{*X*(1) = *y*_1_} × P{*X*(1) = *y*_2_} × … × P{*X*(1) = *y_n_*}.

For convex increasing sequences such as *λ_n_* = *a*(*n* + *b*)*^c^* with *c* > 1, the above probabilities have to be renormalised by dividing by their sum to give a proper probability distribution. A saddlepoint approximation to this sum is available [1, 2] for large *N*:

P{*X*(1) = 0} + P{*X*(1) = 1} + … + P{*X*(1) = *N*} ≈

1 − *Φ*(*w_N_*) − *φ*(*w_N_*) (1/*w_N_* − 1/*u_N_*)

where *w_N_* = sign(*s_N_*) √{ 2(*s_N_* − *K_N_*(*s_N_*))}, *u_N_ = s_N_* √{*K_N_*’’(*s_N_*)}, with *s_N_* the solution of *K_N_’*(*s*) = 1 for *K_N_*(*s*) = − {log(1 − *s*/*λ*_0_) + log(1 − *s*/*λ*_1_) + … + log(1 − *s*/*λ_N_*)}, and *Φ*(.) and *φ*(.) are the standard normal cumulative distribution function and probability density function, respectively. Here, *K_N_*(*s*) is the cumulant generating function of the time taken for the underlying birth process {*X*(*t*); *t* ≥ 0} to increase from 0 to *N*+1. The above expression requires *s_N_* to be non-zero so that *w_N_* and *u_N_* are also non-zero, but as *s_N_* → 0 (1/*w_N_* − 1/*u_N_*) does have the limit *K_N_*’’’(0)/{6 [*K_N_*’’(0)]^3/2^}. Extra terms in the Maclaurin series of (1/*w_N_* − 1/*u_N_*) as a function of *s_N_* will yield improved approximations for small values of *s_N_*.

Note that for large *n*, individual probabilities can also be determined using the above saddlepoint approximation:

P{*X*(1) = *n*} ≈ {1 − *Φ*(*w_n_*) − *φ*(*w_n_*) (1/*w_n_* − 1/*u_n_*)}

− {1 − *Φ*(*w_n_*_-1_) − *φ*(*w_n_*_-1_) (1/*w_n_*_-1_ − 1/*u_n_*_-1_)}.

For practical purposes, exact matrix exponential calculations are quite feasible for values of *N* (or *n*) up to 100 or so, with the saddlepoint approximation used for larger values. For moment calculations and re-normalising the probabilities (if necessary) then *N* of the order of many thousands might be needed for probability distributions with very long tails.

The following MATLAB^®^ code calculates probabilities from the EPPM with *λ_n_* = *a*(*b* + *n*)*^c^* given parameter values *a*, *b* and *c*, for data vector ***y***. From which the (log-)likelihood of the data can be determined and hence maximum likelihood estimates of these parameters.

function p=eppm_probs(a,b,c,y)

%exact calculation of probabilities for n = 0, 1, 2, ..., 100

n=[0:100];

lambda=exp(log(a)+c*log(b+n));

lambda=min(lambda,10^10); %to avoid numerical errors from lambda's too big

Q=-diag(lambda)+diag(lambda(1:length(lambda)-1),1);

p=[1 zeros(1,length(n)-1)]*expm(Q);

%now probabilities for data y

pp=[];

for i=1:length(y);

if y(i)<=max(n) %exact calculation

pp=[pp p(y(i)+1)];

else %saddlepoint approximation

nn=[0:y(i)];

lambda=exp(log(a)+c*log(b+nn));

ppp=saddlepoint(lambda)-saddlepoint(lambda(1:length(lambda)-1));

pp=[pp ppp];

end

end

if c > 1+1e-3

%normalising constant

normc=sum(p);

nn=[0:10000];

lambda=exp(log(a)+c*log(b+nn));

normc=normc+saddlepoint(lambda);

n=[0:100];

lambda=exp(log(a)+c*log(b+n));

normc=normc-saddlepoint(lambda);

else

normc=1;

end

p=pp/normc;

With the following code for the saddlepoint approximation.

function f=saddlepoint(lambda)

%computes saddlepoint approximation for cumulative probabilities

if length(lambda) == 0

f=0;

else

%iterative solution for shat

shat=min(lambda)-1;

shatinc=-(sum(1./(lambda-shat))-1)/sum(1./(lambda-shat).^2);

shat=shat+shatinc;

while abs(shatinc) >= 1e-5

shatinc=-(sum(1./(lambda-shat))-1)/sum(1./(lambda-shat).^2);

shat=shat+shatinc;

end

what=sign(shat)*sqrt(2*(shat*1+sum(log(1-shat./lambda))));

uhat=shat*sqrt(sum(1./(lambda-shat).^2));

if abs(shat) > 1e-2

%exact for shat not close to 0

f=1-0.5*(1+erf(what/sqrt(2)));

f=f-(1/what-1/uhat)*(1/sqrt(2*pi))*exp(-0.5*what^2);

else

%asymptotic approximation for shat close to 0

k2=sum(1./lambda.^2); k3=2*sum(1./lambda.^3);

k4=6*sum(1./lambda.^4); k5=24*sum(1./lambda.^5);

a0=k3/6/k2^(3/2); a1=k4/8/k2^(3/2)-5*(k3^2)/24/k2^(5/2);

a2=k5/20/k2^(3/2)-k3*k4/4/k2^(5/2)+95*(k3^3)/432/k2^(7/2);

f=1-0.5*(1+erf(what/sqrt(2)));

f=f-(1/sqrt(2*pi))*exp(-0.5*what^2)*(a0+a1*shat+a2*shat^2);

end

end

f=min(1,max(0,f)); %keeps f between 0 and 1

**References in the Appendix**

1. Daniels HE. The saddle point approximation for a general birth process. *Journal of Applied Probability*. 1982; 19(1): 20-28.

2. Lugannani R, Rice S. (1980) Saddlepoint approximations for the distribution of the sum of independent random variables. *Advances in Applied Probability*. 1980; 12(2),:475-490.
